# Unsupervised Damage Detection for Offshore Jacket Wind Turbine Foundations Based on an Autoencoder Neural Network

**DOI:** 10.3390/s21103333

**Published:** 2021-05-11

**Authors:** Maria del Cisne Feijóo, Yovana Zambrano, Yolanda Vidal, Christian Tutivén

**Affiliations:** 1 Mechatronics Engineering, Faculty of Mechanical Engineering and Production Science (FIMCP), ESPOL Polytechnic University, Escuela Superior Politécnica del Litoral (ESPOL), Campus Gustavo Galindo Km. 30.5 Vía Perimetral, Guayaquil 09-01-5863, Ecuador; mdfeijoo@espol.edu.ec (M.d.C.F.); ypzambra@espol.edu.ec (Y.Z.); cjtutive@espol.edu.ec (C.T.); 2 Facultad de Ingenierías, Universidad ECOTEC, Km. 13.5 Vía a Samborondón, Samborondón 092302, Ecuador; 3 Control, Modeling, Identification and Applications (CoDAlab), Department of Mathematics, Escola d’Enginyeria de Barcelona Est (EEBE), Universitat Politècnica de Catalunya (UPC), Campus Diagonal-Besós (CDB), Eduard Maristany, 16, 08019 Barcelona, Spain; 4 Institut de Matemàtiques de la UPC—BarcelonaTech (IMTech), Pau Gargallo 14, 08028 Barcelona, Spain

**Keywords:** damage diagnosis, structural health monitoring, offshore wind turbine, offshore foundation, autoencoder

## Abstract

Structural health monitoring for offshore wind turbine foundations is paramount to the further development of offshore fixed wind farms. At present time there are a limited number of foundation designs, the jacket type being the preferred one in large water depths. In this work, a jacket-type foundation damage diagnosis strategy is stated. Normally, most or all the available data are of regular operation, thus methods that focus on the data leading to failures end up using only a small subset of the available data. Furthermore, when there is no historical precedent of a type of fault, those methods cannot be used. In addition, offshore wind turbines work under a wide variety of environmental conditions and regions of operation involving unknown input excitation given by the wind and waves. Taking into account the aforementioned difficulties, the stated strategy in this work is based on an autoencoder neural network model and its contribution is two-fold: (i) the proposed strategy is based only on healthy data, and (ii) it works under different operating and environmental conditions based only on the output vibration data gathered by accelerometer sensors. The proposed strategy has been tested through experimental laboratory tests on a scaled model.

## 1. Introduction

In recent years, there has been great concern about increasing global pollution caused by dependence on non-renewable energy, leading many countries to increase their development of clean renewable energy. Renewable energy is the energy obtained from renewable resources such as wind, sun, and water. Wind energy is one of the most widely accepted alternatives because it is clean (reduces global pollution) and is found in many geographical locations worldwide. The annual report of the Global Wind Energy Council (GWEC) stated that installed wind power capacity had reached 650 GW by the end of 2019, a growth of 10% over the previous year, and new wind power installations surpassed 60 GW, a 19% growth over 2018 [1]. Onshore installations led new wind installations during previous years, but offshore wind energy has attracted worldwide attention because of its many advantages, including substantial energy reserves, faster and steadier wind speeds, and low environmental influence. In 2019, the offshore wind market passed the 6 GW milestone, making up 10% of global new installations, the highest level to date. The 29 GW of cumulative offshore wind power capacity represent 4.5% of the total cumulative installations. With the development of wind power technology, the offshore wind industry has trended towards increased water depths, and increased turbine capacity (with its associated rotor size increase) [2]. However, an important issue that comes with the use of offshore wind turbines (WTs) is that they are subject to extreme environmental conditions caused by wind, waves and currents [3]. They require rigorous safety measures, as it is difficult and costly to perform maintenance and inspection work on these large turbines, mainly due to limitations stemming from their height and remote locations. Moreover, the tower and blades have become more flexible, increasing the importance of the structural safety of WTs under operational conditions. In particular, more than 900 accidents have occurred in the United Kingdom in the last five years, of which 52 were structural failures, the third most common accident cause [4]. The events leading to offshore WT failure are varied and occur in places such as the blades, tower, and foundations [5,6]. To reduce downtime due to unexpected failures, the wind industry uses preventive maintenance (scheduled maintenance) and corrective maintenance (remedy failures) approaches. However, to achieve greater efficiency and reduced maintenance costs, research must evolve to the development of techniques based on the condition of the assets (predictive maintenance) [7]. Therefore, the development of a structural health monitoring (SHM) strategy is imperative for offshore WT structures.

**Figure 1:**
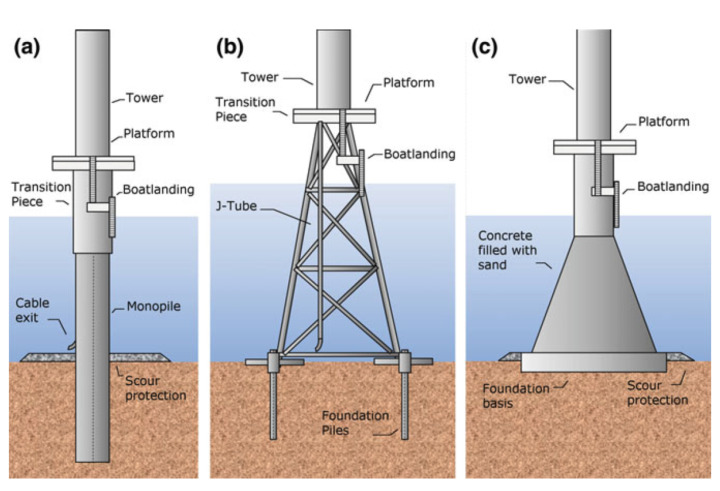
Fixed-type WT foundations [9]. Monopile (**a**), jacket (**b**), and gravity-based (**c**).

Offshore fixed WTs have their foundations on the seabed. There are various types of offshore foundations, depending on the depth at which the WT is installed. The most common foundations are monopile, jacket, and gravity-based structures [8] (see Figure 1). Monopile foundations are used at depths of up to 15 m, gravity foundations are preferred at depths of up to 30 m, and jackets are the preferred option at greater depths [9].

To avoid the collapse of the entire structure, it is important to detect foundation damage at an early stage. As stated in “Long-term research challenges in wind energy—a research agenda by the European Academy of Wind Energy” [10]:

A defining marine environment main characteristic is that structures are always subject to excitations. Techniques for structural health monitoring, vibration, and data analysis must be capable of coping with such ambient excitations. As the input is typically not known, a normal input-output formalism cannot be used.

Thus, to overcome the challenge posed by the fact that the only excitation of the WT is assumed to be caused by wind, which is typically not known, an SHM strategy for jacket-type foundations is given in this study based on a vibration-response-only methodology. In recent years, interest in this type of methodology has grown because the excitation in many applications cannot be imposed and often cannot be measured. For example, in [11], an SHM method for floating offshore WTs was suggested and tested using operational modal analysis with numerical-sensor signals. The results showed that the curvature mode shape was the most effective modal property to detect damage location and intensity. Likewise, Mieloszyk et al. [12] contributed an SHM system for real tripod WT based on Fiber Bragg Grating (FBG) sensors to detect and localize underwater damages of WT support structure. Furthermore, in [13], a random coefficient Gaussian mixture model (GMM-RC) was adopted and evaluated based on the analysis of vibration response signals, resulting in significant performance improvement. A meaningful work was presented by Fushun et al. [14], who proposed a new time-frequency analysis method based on single mode function decomposition to overcome the mode-mixing problem in the SHM of offshore WTs. Finally, ref. [15] presents a vibration-response-only approach via convolutional neural networks and [16] proposes the fractal dimension as a suitable feature to identify and classify different types of damage.

The works mentioned above require historical damage data. In particular, historical offshore WT foundation damage data must be accurately labeled with the type of damage (in this case, fatigue cracks introduced at different bars). Obtaining this real data is difficult or even nearly impossible, and, furthermore, it is difficult to obtain the correct labels for each damage type. It is time-consuming, error-prone, and likely to result in a set of labeled vectors that has an unbalanced number of classes. In contrast, in this study, historical damage data are not needed; thus, the proposed strategy can be applied to any offshore wind farm jacket-type foundations, even if damage data have not yet been recorded. Specifically, a normal behavior model is proposed in this work, i.e., the model is built using only normal (healthy) operational data. The proposed method can be summarized in the following steps: (i) wind excitation is simulated as Gaussian white noise, and the data from the WT are collected using a set of accelerometers; (ii) the raw data are preprocessed; (iii) an autoencoder neural network classifies the damage. The damage detection strategy is applied to 5 mm crack damage for different jacket WT structure bars and different wind excitation levels. The results obtained demonstrate the reliability of the proposed approach.

The remainder of this paper is organized as follows. The experimental set-up is shown in Section 2. The stated damage detection methodology is comprehensively explained in Section 3. The results are presented and discussed in Section 4. Finally, conclusions are drawn in Section 5.

## 2. Experimental Set-Up

**Figure 2:**
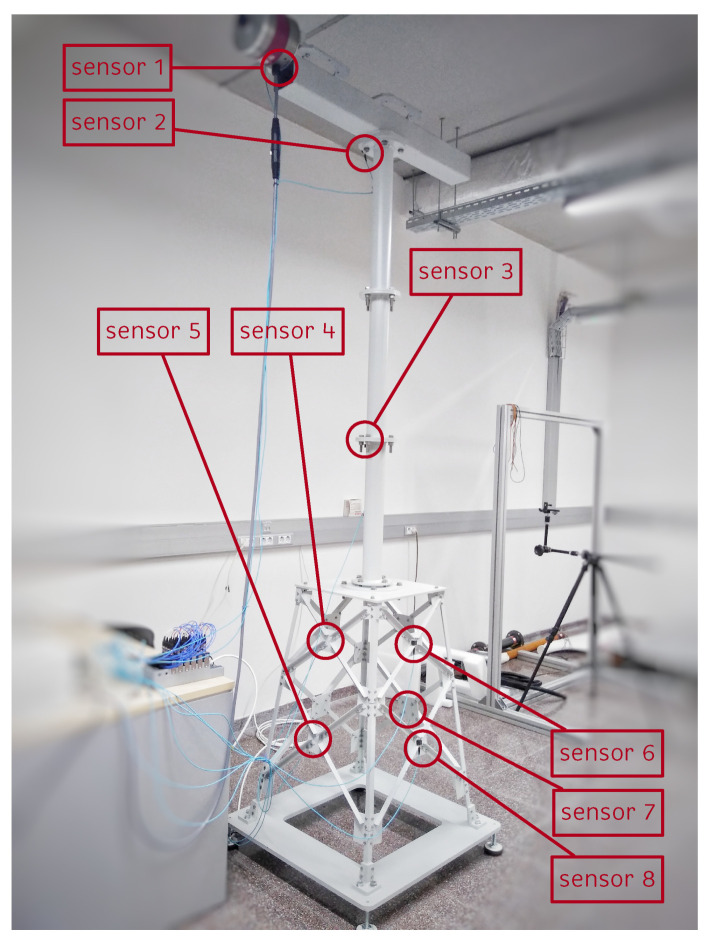
Location of the sensors (accelerometers) in the structure [19].

In this study, the downscaled replica of an offshore WT shown in Figure 2 is used. The structure measures 2.7 m high and is composed of three main parts: the jacket, the tower, and the nacelle. The last part is simulated using a beam that supports an inertial shaker (model GW-IV47), which simulates the environmental effects of wind on the rotor. The electrical signal applied to the shaker is provided by a function generator (model GW INSTEK AF-2005) that produces a white noise (WN) signal, which passes through an amplifier (model PA300E) and is then sent to the inertial shaker.

In general terms, WTs have three operational regions [17]. First, in Region 1, when the wind speed is low, the power available in the wind is lower than the losses in the turbine system; hence, the turbines are not run. In Region 2, the partial load region, the wind is below the rated wind speed. The turbine is controlled to maximize the power captured by adjusting the generator torque to obtain an optimum ratio between the tip speed of the blades and the wind speed. Finally, in Region 3, the full load region, the wind speed is greater than the rated wind speed, so the main task of the controller is to adapt the aerodynamic efficiency of the rotor by pitching the blades into or out of the wind to keep the rotor speed at its rated value. To account for these operational regions, three different amplitudes with factors of 0.5, 1, and 2 are used as the input signal to the shaker.

To analyze the dynamic behavior of the structure, eight triaxial accelerometers (PIEZOTRONIC model 356A17) are used, of which five are placed in the jacket, one in the tower, and the remaining two in the nacelle, as shown in Figure 2. The method used to find the optimal location and number of these sensors is described in [18].

**Figure 3:**
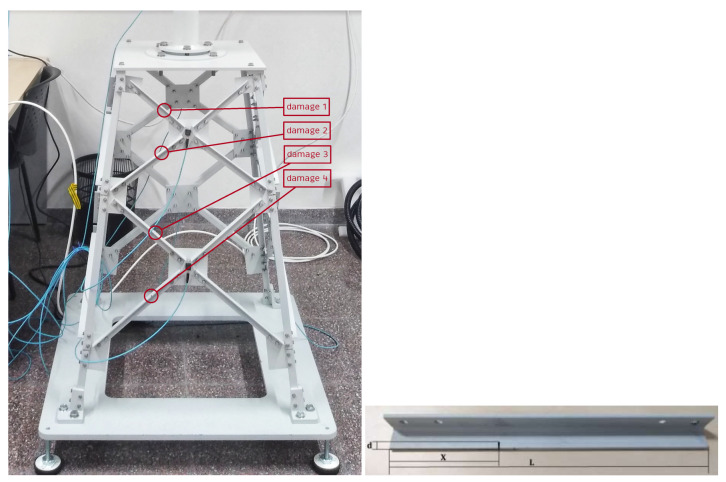
Damage location in the different levels of the jacket structure (**left**), [19]. Crack damage where *L* is the length of the bar, *d* = 5 mm is the crack size, and X=L/3 is the location of the crack in the bar (**right**).

The jacket foundation is composed of 32 steel bars bolted together. The nuts used are self-locking with washers to prevent them from loosening due to vibration. All joints are tightened to 12 Nm. As shown in Figure 3 (left), the jacket has four different lengths of bars, which are used at different depths. Please note that Level 1, the shortest bar, is used closest to the sea surface. Longer bars are used at deeper locations.

The most common damage in offshore foundations is caused by corrosion-fatigue cracks of the structure, see [20,21]. The probability of detection of a fatigue crack is low for small crack sizes. However, for larger and therefore better detectable fatigue cracks, the crack growth rate accelerates rapidly [22]. Consequently, there is only a small time window for detection and repair of this type of cracks before failure. Thus, in this work, a 5 mm crack is considered located at different bars of the jacket structure, one at a time, as shown in Figure 3 and Table 1. Please note that in [18] a modal analysis and power spectral density signal processing methods were not able to detect this 5 mm crack located in the substructure using a similar laboratory tower model.

For data acquisition, six National Instruments input modules (model NI 9234) are used, which are placed in a chassis (model cDAQ). This allows the processing of the signals received from the accelerometers.

## 3. Damage Detection Methodology

**Figure 4:**
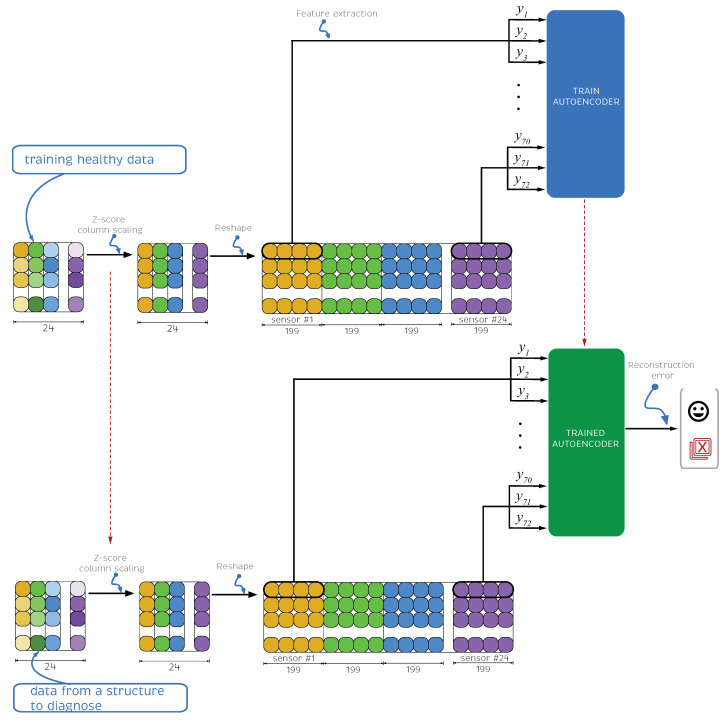
Flowchart of the stated damage detection methodology.

In this section, the damage detection methodology is comprehensively stated and its flowchart is shown in Figure 4. First, the experimental data are collected for different structural states: healthy, and the structure with a cracked bar at four different locations. Second, the data are split into train, validation and test sets. It is noteworthy that the train and validation data only contain healthy samples. Then, the test dataset will contain all possible states: healthy samples, and damaged samples associated with the four studied damage scenarios. The reason that the train and validation sets only contain healthy data is that the autoencoder model will be trained to reproduce at the output the same values used as input (with some associated error noted here as the reconstruction error) only with healthy data. Then, when testing, in case a damage sample is used as input, then the trained model will not be able to properly reconstruct the outputs from the inputs, i.e., a higher reconstruction error will be obtained. In this manner, the trained model is a so-called normality model. It only needs normal (healthy) data to be trained. Then, when data that does not follow normality is used as input, the autoencoder will not be able to reconstruct with the same fidelity the outputs and the highest reconstruction error will trigger an alarm advising that an abnormal sample has been found. Third, the data are normalized using Z-score column scaling. Fourth, and crucial step, is feature engineering to reshape the data in such a way that each sample contains information from different consecutive time steps of each sensor and finally design the features that will be used as inputs. Fifth, the autoencoder neural network structure, optimization method, loss function, and associated hyperparameters are stated. Sixth, and final step, a damage indicator is proposed based on the reconstruction error. When its value is greater than a prescribed threshold, an alarm is triggered to warn of a possible damage.

### 3.1. Data Collection

In total, 105 experimental tests were carried out: 45 with the healthy structure and the remaining 60 simulating different damage types, which are detailed in Table 1. Furthermore, as mentioned above, the laboratory experimental tower operated in the three different wind regions, with tests carried out for each one with four different damage types. Table 2 lists the number of experiments performed at different WN input amplitudes in each of the structural states.

As mentioned at the beginning of this section, the accelerometers used are triaxial, i.e., each of these sensors collects data along three axes. Therefore, the total number of sensors considered in this study is *N* = 8 × 3 = 24 sensors.

The data acquisition of the structure in each experimental test was carried out for 60 seconds with a sampling frequency of approximately 275.28 Hz, i.e., in each experiment, 275.28 [Hz] (approximately) × 60 [s] = 16,517 data measurements were obtained for each of the 24 sensors. Thus, in each experiment a data matrix was obtained with the following structure:

(1) $$\left(\left.\begin{array}{cccc} x_{11} & x_{12} & \cdots & x_{1N}\\ \vdots & \vdots & \ddots & \vdots \\ x_{i1} & x_{i2} & \cdots & x_{iN}\\ \vdots & \vdots & \ddots & \vdots \\ x_{m1} & x_{m2} & \cdots & x_{mN} \end{array}\right.\right)\in M_{m\times N}\left(R\right),$$

where *m* = 16,517 is the number of samples taken in one experimental test, *N* is the number of sensors, and M(m×N) corresponds to the vector space of (m×N)-dimension matrices over R. In particular, $x_{ij}$ denotes the measurement related to sensor number *i* at time step *j* of the undergoing experimental test.

Stacking the matrices obtained in the experimental tests according to their structural state, five different matrices (corresponding to the five different structural states) were obtained, denoted $X^{0},X^{1},X^{2},X^{3},$ and $X^{4}$. The first matrix contains data from 45 experiments performed with the healthy structure, and the remaining four matrices contain data from 15 experiments, each performed with the damaged structure simulating jacket-bar crack damage at levels 1, 2, 3, and 4. The matrices obtained have the following structure:

(2) $$\begin{array}{c} \left(\begin{array}{cccc} x_{11}^{\left(1\right)} & x_{12}^{\left(1\right)} & \cdots & x_{1N}^{\left(1\right)}\\ \vdots & \vdots & \ddots & \vdots \\ x_{m1}^{\left(1\right)} & x_{m2}^{\left(1\right)} & \cdots & x_{mN}^{\left(1\right)}\\ \; & \; & & \\ \; & \; & & \\ x_{11}^{\left(2\right)} & x_{12}^{\left(2\right)} & \cdots & x_{1N}^{\left(2\right)}\\ \vdots & \vdots & \ddots & \vdots \\ x_{m1}^{\left(2\right)} & x_{m2}^{\left(2\right)} & \cdots & x_{mN}^{\left(2\right)}\\ \; & \; & & \\ \; & \; & & \\ \vdots & \vdots & \ddots & \vdots \\ \; & \; & & \\ \; & \; & & \\ x_{11}^{\left(L\right)} & x_{12}^{\left(L\right)} & \cdots & x_{1N}^{\left(L\right)}\\ \vdots & \vdots & \ddots & \vdots \\ x_{m1}^{\left(L\right)} & x_{m2}^{\left(L\right)} & \cdots & x_{mN}^{\left(L\right)} \end{array}\right)\; \end{array}$$

where the superscript indicates the experiment number. The number of rows in the new matrix corresponds to the number of samples, *m*, taken in an experiment multiplied by the number of experiments performed, *L*, and the number of columns corresponds to the number of sensors used, *N*.

Table 3 shows the characteristics and dimensionality of the five matrices. It is important to note that the number of rows could vary among matrices. However, the number of columns in all matrices must be strictly the same, because the number of sensors used in the experimental tests must be the same.

To train the WT damage diagnosis model in this study, only the data collected from the healthy WT are used. Once the training process is finished, the model is tested using the matrices $X^{1}$, $X^{2}$, $X^{3}$, and $X^{4}$, which contain the data collected with the WT simulating the four types of damage described above, together with a subset of the healthy data matrix $X^{0}$. The split of matrix $X^{0}$ into the training, validation, and testing sets is detailed in the next subsection.

### 3.2. Data Split: Train, Validation and Test Sets

To train the neural network model, only healthy data are used. Recall that the matrix $X^{0}$ contains the healthy samples. This matrix is split into three different subsets of data: training $X_{train}^{0}$, validation $X_{val}^{0}$, and testing $X_{test}^{0}$ [23] using the following percentages:

- Training set: 70%
- Validation set: 15%
- Test set: 15%

These three sets allow the precision of the model to be evaluated. The training set is used to fit the model, i.e., the model learns these data. The validation set is used to impartially evaluate the training of the model, in addition to allowing modification of the hyperparameters to avoid overfitting. Finally, the test set is used once the fully trained model has been obtained, using said data for a final evaluation of the precision of the model on unseen data.

Table 4 describes the dimensionality of the three different subsets.

### 3.3. Data Preprocess

In machine and deep learning, data preprocessing is one of the most important steps. Skilled data preprocessing can make the difference between a successful or unsuccessful model. Generally, this stage is divided into the following four steps [24]:

- Data cleaning
- Normalization
- Feature engineering
- Imbalanced data management

On the one hand, in this project, all experimental tests were carried out under controlled situations within a laboratory, so the information obtained does not present the common problems and errors found in real-world databases, such as missing data, noise, and outliers. On the other hand, because the approach is unsupervised and only healthy data are used to train the model, the inconvenience of data imbalance is avoided. That is why the data cleansing and unbalanced data management steps are not required in this work.

#### 3.3.1. Normalization

Normalization is a widely used technique in data preprocessing. It consists of transforming the data to a common scale [25], ensuring that the contribution of each feature is equally important, which in many cases improves the performance of the model. In this work, as in [26], Z-score column scaling is used, i.e., each column of matrix $X_{train}^{0}$ is scaled to have zero mean and standard deviation equal to one. The calculated statistics from $X_{train}^{0}$ (mean and standard deviation of all measurements in each column) are employed to scale all data, i.e., the other matrices: $X_{val}^{0}$, $X_{test}^{0}$, $X^{1}$, $X^{2}$, $X^{3}$, and $X^{4}$.

#### 3.3.2. Feature Engineering

Feature engineering is a data preprocessing step that creates new features from existing ones [27]. In this study, this step has been divided into two parts: first, data are reshaped, and then new features are extracted.

By analyzing all the matrices obtained, it can be inferred that the amount of information obtained from each sensor in each sample (row) may be scarce or insufficient because only one time stamp for data from each sensor is available for each sample. Therefore, to obtain the most complete information, which also contains the temporal evolution of each sensor, the original matrix is reshaped to increase the amount of information available in each sample. In this process, each matrix is reshaped by relocating every κ= 199 rows of the matrix into columns:

(3) $$\left[\begin{array}{cccccccccc} x_{1,1}^{\left(1\right)} & \cdots & x_{\kappa ,1}^{\left(1\right)} & x_{1,2}^{\left(1\right)} & \cdots & x_{\kappa ,2}^{\left(1\right)} & \cdots & x_{1,N}^{\left(1\right)} & \cdots & x_{\kappa ,N}^{\left(1\right)}\\ x_{\kappa +1,1}^{\left(1\right)} & \cdots & x_{2\kappa ,1}^{\left(1\right)} & x_{\kappa +1,2}^{\left(1\right)} & \cdots & x_{2\kappa ,2}^{\left(1\right)} & \cdots & x_{\kappa +1,N}^{\left(1\right)} & \cdots & x_{2\kappa ,N}^{\left(1\right)}\\ \vdots & \ddots & \vdots & \vdots & \ddots & \vdots & \ddots & \vdots & \ddots & \vdots \\ x_{m-\kappa +1,1}^{\left(1\right)} & \cdots & x_{m,1}^{\left(1\right)} & x_{m-\kappa +1,2}^{\left(1\right)} & \cdots & x_{m,2}^{\left(1\right)} & \cdots & x_{m-\kappa +1,N}^{\left(1\right)} & \cdots & x_{m,N}^{\left(1\right)}\\ x_{1,1}^{\left(2\right)} & \cdots & x_{\kappa ,1}^{\left(2\right)} & x_{1,2}^{\left(2\right)} & \cdots & x_{\kappa ,2}^{\left(2\right)} & \cdots & x_{1,N}^{\left(2\right)} & \cdots & x_{\kappa ,N}^{\left(2\right)}\\ x_{\kappa +1,1}^{\left(2\right)} & \cdots & x_{2\kappa ,1}^{\left(2\right)} & x_{\kappa +1,2}^{\left(2\right)} & \cdots & x_{2\kappa ,2}^{\left(2\right)} & \cdots & x_{\kappa +1,N}^{\left(2\right)} & \cdots & x_{2\kappa ,N}^{\left(2\right)}\\ \vdots & \ddots & \vdots & \vdots & \ddots & \vdots & \ddots & \vdots & \ddots & \vdots \\ x_{m-\kappa +1,1}^{\left(2\right)} & \cdots & x_{m,1}^{\left(2\right)} & x_{m-\kappa +1,2}^{\left(2\right)} & \cdots & x_{m,2}^{\left(2\right)} & \cdots & x_{m-\kappa +1,N}^{\left(2\right)} & \cdots & x_{m,N}^{\left(2\right)}\\ \vdots & \ddots & \vdots & \vdots & \ddots & \vdots & \ddots & \vdots & \ddots & \vdots \\ x_{1,1}^{\left(L\right)} & \cdots & x_{\kappa ,1}^{\left(L\right)} & x_{1,2}^{\left(L\right)} & \cdots & x_{\kappa ,2}^{\left(L\right)} & \cdots & x_{1,N}^{\left(L\right)} & \cdots & x_{\kappa ,N}^{\left(L\right)}\\ x_{\kappa +1,1}^{\left(L\right)} & \cdots & x_{2\kappa ,1}^{\left(L\right)} & x_{\kappa +1,2}^{\left(L\right)} & \cdots & x_{2\kappa ,2}^{\left(L\right)} & \cdots & x_{\kappa +1,N}^{\left(L\right)} & \cdots & x_{2\kappa ,N}^{\left(L\right)}\\ \vdots & \ddots & \vdots & \vdots & \ddots & \vdots & \ddots & \vdots & \ddots & \vdots \\ x_{m-\kappa +1,1}^{\left(L\right)} & \cdots & x_{m,1}^{\left(L\right)} & x_{m-\kappa +1,2}^{\left(L\right)} & \cdots & x_{m,2}^{\left(L\right)} & \cdots & x_{m-\kappa +1,N}^{\left(L\right)} & \cdots & x_{m,N}^{\left(L\right)} \end{array}\right]$$

Table 5 shows the new dimensions of the matrices after the reshaping has been carried out to increase the amount of information per sample (row) for each sensor.

To improve and facilitate the training of the model in this study, three features are selected for each sensor: the mean (μ), standard deviation (σ), and instantaneous spectral entropy (*H*), which are detailed in the next section.

### 3.4. Autoencoder Inputs

The autoencoder neural network (ADNN) inputs, or also known as input nodes, are present in the first layer of the ADNN and are the only nodes that provide information from the outside world to the network. In this study, as mentioned in the previous section, the autoencoder-selected inputs are the mean, standard deviation, and instantaneous spectral entropy for each sample and for each sensor. Next, a brief review of the instantaneous spectral entropy is given.

The instantaneous spectral entropy of a signal measures the spectral power distribution in the frequency domain, treating it as a probability distribution and computing its Shannon entropy. The Shannon entropy has been proven to be useful to extract features for fault detection and diagnosis, see [28,29]. The equations to compute the instantaneous spectral entropy are given in [30] and summarized here as follows. For a discrete signal, x(i), also its discrete time-frequency power spectrogram $S_{x}\left(t,f\right)$ is first computed. Then, the probability distribution at time *t*, P(t,j), is given by

$$P\left(t,j\right)=\frac{S_{x}\left(t,j\right)}{\sum_{f}S_{x}\left(t,f\right)}.$$

Finally, the instantaneous spectral entropy at time *t*, H(t), can be written as

$$H\left(t\right)=-\sum_{j=1}^{J}P\left(t,j\right)\log_{2}P\left(t,j\right),$$

where *J* is the total number of frequency points.

### 3.5. Autoencoder Architecture

An autoencoder is a neural network model based on unsupervised learning algorithms, which allows the efficient compression (encoding) and decompression (decoding) of data. Its main objective is to reproduce the input vector in the output vector, based on the characteristics obtained in the network training [31]. In this study, data from the healthy matrix is used to train the autoencoder. Then, when testing, if a new healthy sample is used as the input, the ADNN can replicate the input. On the other hand, when a new sample related to damaged data is used as the input, the model will not be capable of successfully reconstructing the sample. Thus, a higher error between input and output will trigger an alarm about initial damage in the structure.

**Figure 5:**
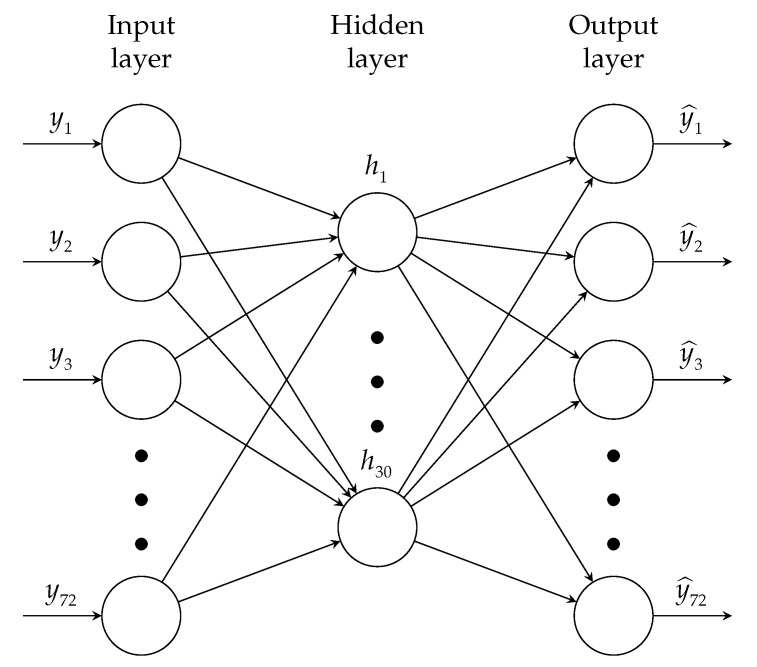
Autoencoder proposed architecture. There are 3 inputs (mean, standard deviation, and entropy) for each one of the 24 sensors giving a total of 72 inputs. The hidden layer is set to 30 nodes.

The architecture of the proposed ADNN is composed of three layers: the input, hidden, and output layers. The first layer, denoted $l_{1}$, is the input layer. Its information is encoded in the hidden layer, $l_{2}$, and then decoded in the output layer, $l_{3}$. Each layer consists of a predefined number of nodes. In the input and output layers, the number of nodes is 72, and in the hidden layer, 30. Figure 5 shows the ADNN architecture.

The activation functions used for the encoder and decoder are the logistic sigmoid and purelin functions, respectively. For the output layer a linear function is used, since it is not necessary to scale the data within a specific range, thus the function used in this case is the purelin or linear transfer function. In addition, the cost function, or loss function, used is the mean squared error (MSE). The scaled conjugate gradient (SCG) is used as the optimization method. It calculates the second-order information from the two first-order gradients of the parameters using all the training data [32]. Unlike other optimizers, this algorithm does not perform a line search in each iteration to make it more computationally efficient [33]. More detailed information about this algorithm can be found in [34]. Finally, a summary of the selected hyperparameters for the model can be seen in Table 6.

### 3.6. Damage Detection Indicator

To determine the behavior of the network with new and unknown data, it is necessary to determine an indicator that through the analysis of the input nodes and the output nodes of the network, allows identification of the healthy data from the unhealthy data.

The indicator used in this study is the reconstruction error, RE, defined as:

(4) $$RE\left(y\right)=||y-\hat{y}{\left|\right|}^{2}.$$

where y represents the input layer values and $\hat{y}$ the output layer approximation.

When the data are healthy, the network can reconstruct the data as closely as possible to their original values because they maintain a similar distribution as the data with which the network was trained. Therefore, the value of RE should be small. On the other hand, if the data belong to a damaged state, the network will not be able to rebuild the output as accurately, and RE will be higher. In other words, the reconstruction error is used as an indicator of a damaged state.

As mentioned in Section 3.1, data are collected from trials conducted at different amplitudes. It is noteworthy that a single model is proposed for all these amplitudes. However, to achieve a better damage detection strategy, a specific threshold is determined for each of the amplitudes. To determine the value of the thresholds, it is assumed that the RE signal follows a normal distribution and that most of the data are within 99.7% of the curve, which corresponds to three standard deviations. Therefore, the thresholds are defined as shown in Table 7, where μ and σ correspond to the mean and standard deviation of the RE values, respectively, of the training and validation sets for each amplitude.

Finally, it is important to note that in real application, the reconstruction error could also arise due to other causes not related to damage as measurement errors or faulty sensors.

## 4. Results

In this section, the results of the proposed methodology for damage diagnosis for offshore jacket WT foundations are presented and analyzed.

**Figure 6:**
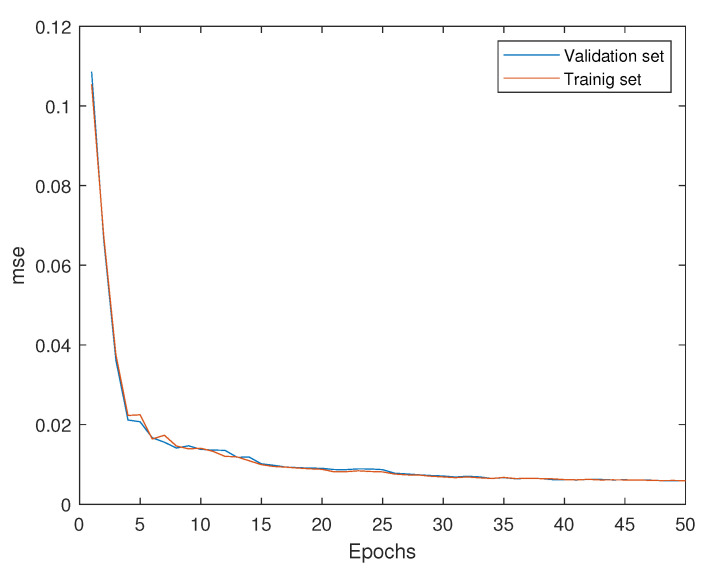
Mean squared error of training and validation data sets.

In Figure 6, the network training process is shown.

It is observed that the MSE of both the training and validation sets decreases in each epoch, reaching the best performance in epoch 50 with a value of 0.007. Recall that healthy data from all amplitudes is used to train a single model, i.e., a unique model is trained to cope with the various regions of operation (different wind speeds) of the WT simulated there with different amplitudes (with factors of 0.5, 1, and 2) of the white noise signal used as input to the shaker.

**Figure 7:**
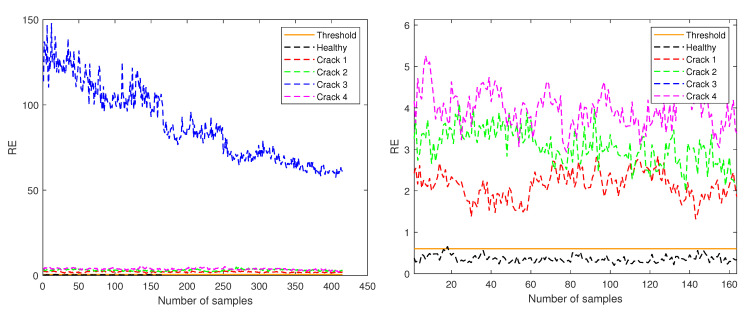
RE for test samples corresponding to amplitude 0.5 (**left**) and zoom in the region with y-axis from 0 to 6 (**right**).

**Figure 8:**
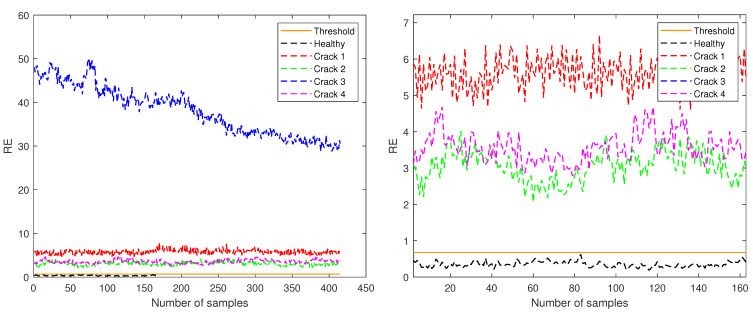
RE for test samples corresponding to amplitude 1 (**left**) and zoom in the region with y-axis from 0 to 7 (**right**).

**Figure 9:**
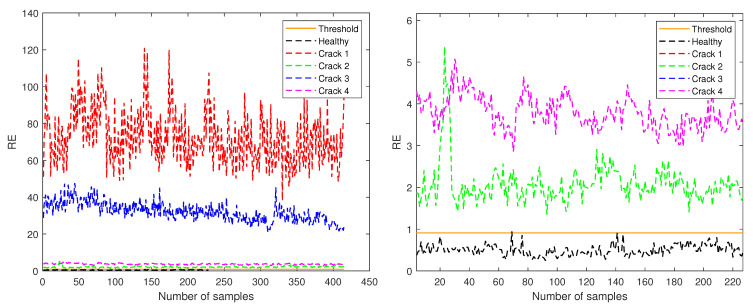
RE for test samples corresponding to amplitude 2 (**left**) and zoom in the region with y-axis from 0 to 6 (**right**).

Figure 7, Figure 8 and Figure 9 show the RE values for each of the samples in the five different structural states (detailed in Table 1) test sets according to their amplitude. Furthermore, the orange continuous line indicates the threshold established to determine and classify healthy and unhealthy samples.

In Figure 7, only the data corresponding to amplitude 0.5 are shown, and the threshold is indicated at the RE value of 0.6017. In this case, the crack at level 3 has a RE one order of magnitude higher than the other cracks and the healthy case as can be seen in the left plot of Figure 7. When the plot is zoomed into the region with y-axis from 0 to 6 (right plot of Figure 7) it can be seen that cracks at levels 1, 3 and 4 have RE of the same magnitude, all of them being higher than the threshold value. Only a few samples from the test healthy data have RE values above the defined threshold.

In Figure 8, only the data for amplitude 1 are plotted and the threshold value is marked at 0.6759. In this case, again, the crack at level 3 has the higher RE. In this case, not a single sample of the test healthy data trespasses the threshold.

Finally, Figure 9 corresponds to amplitude 2, and the threshold is shown at 0.9119. In this case, cracks at level 1 and 3 have higher RE values. When the plot is zoomed in the region with y-axis from 0 to 6 (right plot of Figure 9) it can be seen that cracks at levels 2 and 4 have RE of the same magnitude, all of them being higher than the threshold value. Only a few samples from the test healthy data have RE values above the defined threshold. This case, related to the higher wind speed region, is the one where the RE distributions differ more among the different cracks and, in the case a supervised approach could be used, it would be straightforward to localize the damage.

Table 8 shows the confusion matrix over the test sets. The confusion matrix contains the number of true healthy samples correctly predicted (true negative, TN), the true healthy samples wrongly predicted as damage (false positive, FP), the true damage samples wrongly predicted (false negative, FN), and the number of true damage samples correctly predicted (true positive, TP). It can be seen that the model correctly classifies all the samples with damage, while also correctly classifying 99.5% of the healthy samples, failing in only three samples (false positive).

A selection of performance indicators is used to track the results, namely: accuracy, precision, recall, F1 score, and specificity. Their definitions are briefly recalled hereby,

$$\mathrm{Accuracy}=\frac{TP+TN}{TP+TN+FP+FN},$$

$$\mathrm{Precision}=\frac{TP}{TP+FP},$$

$$\mathrm{Recall}=\frac{TP}{TP+FN},$$

$$F1=\frac{TP}{TP+\frac{1}{2}\left(FN+FP\right)},$$

$$\mathrm{Specificity}=\frac{TN}{TN+FP}.$$

From the information in Table 8, the proposed fault detection method achieves an accuracy of 99.8%, a precision of 99.7%, a recall of 100%, an F1-score of 99.8%, and finally a specificity of 99.4%.

In a nutshell, the results manifest that the stated fault detection method is promising as it achieved great performance on different test sets including various crack damaged bars at different locations as well as healthy data.

In real-life applications, during long-term monitoring, the proposed approach can distinguish between natural variations of the dynamic behavior of the wind turbine (for example, due to wear of the turbine) and true damage based on the reconstruction error evolution over time. In particular, an abrupt change of the reconstruction error must trigger an alarm. However, the slow change dynamics, or drift, of the reconstruction error over years should be considered to be normal. This drift of the reconstruction error over years may lead to reconsidering defining the threshold values using data from recent years.

Finally, it is noteworthy that environmental and operational conditions (EOC) are crucial when handling long-term monitoring, as they can impede damage detection. Large fluctuations in EOCs make EOC monitoring almost as important as structural monitoring itself. Thus, its influence should be taken into account. Several methods for EOC compensation for WTs have been developed to make SHM possible. For example, in [35] affinity propagation clustering is used to delineate data into WT groups of similar EOC. However, this work is an experimental proof of concept and EOC compensation is left as future work using pattern recognition techniques in a more realistic environment.

## 5. Conclusions

In this work, an advance damage detection method system was proposed and proven to detect a 5 mm crack in different jacket structure levels. In particular, a damage detection methodology that requires only healthy data and where the excitation given by the wind is not known is deployed. Furthermore, the stated strategy works under different operational conditions to which WTs are subject, including all three WT operational regions. The conceived damage detection methodology performed at very close to an ideal level, achieving an accuracy of 99.8%, a precision of 99.7%, and a recall of 100%. The obtained results evidence that the proposed autoencoder neural network is promising as SHM strategy for offshore WT foundations.

In a nutshell, the advantages of the proposed damage detection methodology that should be highlighted are:

- There is no need for historical faulty data.
- It works under all regions of operation of the wind turbine.
- It is based only on the output vibration data gathered by accelerometer sensors (the excitation given by the wind is assumed to be unknown). Thus, it is a vibration-response-only methodology.
- During long-term monitoring, the proposed approach can distinguish between natural variations of the dynamic behavior of the wind turbine (for example, due to wear of the turbine) and true damage based on the reconstruction error evolution over time (redefining the threshold values using data from recent years).
- The performance indicators show all results above 99.4%.

On the other hand, the main disadvantage that will be faced as immediate future work is that it is needed to validate the proposed strategy in a more realistic environment that takes into account various environmental conditions by placing the laboratory tower in a water tank facility subjecting it to the action of regular and irregular waves and using pattern recognition techniques.

Furthermore, three numerical models and one real scale laboratory tower are studied in reference [18], where the correspondence between the 5 mm studied crack and real-size cracks is established, as well as the correspondence of the acceleration obtained in the scaled experiment and the full-size wind turbine acceleration. In our work, only the real scale laboratory tower is used. Although the next step is to validate the proposed approach in the full-scale numerical models (to have a preliminary outcome on how the scale can affect the results), it is expected that the performance of the methodology will not be affected based on related previous research given in reference [18].

Finally, other neural networks, such as convolutional neural networks with a support vector machine after the feature compressed layer and a convolutional autoencoder, should be studied, as they have interesting properties that initially make them appropriate for the problem under consideration.

## Figures and Tables

**Table 1:** Location of the damaged bars in the jacket foundation.

Damage	Localization
Crack 1	Level 1
Crack 2	Level 2
Crack 3	Level 3
Crack 4	Level 4

**Table 2:** Number of experiments performed for the different WN amplitudes in each of the structural states studied.

Level	Structural State	0.5 WN	1 WN	2 WN	Total Test
	Healthy	15	15	15	45
1	Crack	5	5	5	15
2	Crack	5	5	5	15
3	Crack	5	5	5	15
4	Crack	5	5	5	15

**Table 3:** Dimensionalities of the matrices obtained for each structural state.

Structural State	Matrix	L	Matrix Dimensionality
Healthy	$X^{0}$	45	743,265 × 24
Crack 1	$X^{1}$	15	247,755 × 24
Crack 2	$X^{2}$	15	247,755 × 24
Crack 3	$X^{3}$	15	247,755 × 24
Crack 4	$X^{4}$	15	247,755 × 24

**Table 4:** Matrix dimensionality

Structural State	Set	Matrix	Matrix Dimensionality
Healthy	Training	$X_{train}^{0}$	520,285 × 24
Healthy	Validation	$X_{val}^{0}$	111,490 × 24
Healthy	Test	$X_{test}^{0}$	111,490 × 24

**Table 5:** New dimensions of the reshaped matrices.

Original Matrix	Reshaped Matrix	New Dimension
$X_{train}^{0}$	${\hat{X}}_{train}^{0}$	2614 × 4776
$X_{val}^{0}$	${\hat{X}}_{val}^{0}$	560 × 4776
$X_{test}^{0}$	${\hat{X}}_{test}^{0}$	560 × 4776
$X^{1}$	${\hat{X}}^{1}$	1245 × 4776
$X^{2}$	${\hat{X}}^{2}$	1245 × 4776
$X^{3}$	${\hat{X}}^{3}$	1245 × 4776
$X^{4}$	${\hat{X}}^{4}$	1245 × 4776

**Table 6:** Hyperparameter configuration for network training.

Hyperparameter	Value
Number of epochs	50
Number of layers	3
Number of nodes in input layer	72
Number of nodes in hidden layer	30
Number of nodes in output layer	72
Loss function	MSE
Activation functions in encoder	Logistic sigmoid
Activation functions in decoder	Purelin
Optimizer	SCG

**Table 7:** Threshold value for each amplitude. Please note that $\mu_{0}$, $\mu_{1}$, and $\mu_{2}$ are the mean values of the RE values for amplitudes 0.5, 1, and 2, respectively. In addition, $\sigma_{0}$, $\sigma_{1}$, and $\sigma_{2}$ are the standard deviation of the RE values for amplitudes 0.5, 1, and 2, respectively.

Amplitude	Threshold
0.5	$\mu_{0}+3\sigma_{0}=0.6017$
1	$\mu_{1}+3\sigma_{1}=0.6759$
2	$\mu_{2}+3\sigma_{2}=0.9119$

**Table 8:** Confusion matrix for binary classification performance assessment over all test sets. Please note that TN stands for true negative, FP stands for false positive, FN stands for false negative, and TP stands for true positive.

		Predicted Class	
		Healthy	Damage
	Healthy	TN=557	FP=3
True class	Damage	FN=0	TP=1245

## Data Availability

The data presented in this work are available on request from the corresponding author.
